# Supplementary material for: Integrated photonic encoder for low power and high-speed image processing
Source: Nat Commun. 2024 May 27;15:4510. doi: 10.1038/s41467-024-48099-2 (PMC11130346; doi:10.1038/s41467-024-48099-2)
Supplement: Supplementary file 1 — Supplementary Information [file 41467_2024_48099_MOESM1_ESM.pdf]

SUPPLEMENTARY INFORMATION

Integrated Photonic Encoder for Low Power and High- Speed Image Processing

Xiao Wang [1, +], Brandon Redding [2, +], Nicholas Karl [3], Christopher Long [3], Zheyuan Zhu [4], James Skowronek [1], Shuo Pang [4], David Brady[1, #], Raktim Sarma[3, 5, *]

[+]Equal contribution

[#]djbrady@arizona.edu

[*]rsarma@sandia.gov

[1] Wyant College of Optical Sciences, University of Arizona, Tucson, Arizona, USA

[2] U.S. Naval Research Laboratory, Washington, DC, USA

[3]Sandia National Laboratories, Albuquerque, New Mexico, USA

[4]CREOL, The College of Optics and Photonics, University of Central Floria, Orlando, Florida, USA

[5]Center for Integrated Nanotechnologies, Sandia National Laboratories, Albuquerque, New Mexico, USA

S1. Additional Experimental results: Compressed and reconstructed images and their statistics.

In the main text, we presented only one example of experimentally compressed and reconstructed image. In Fig S1, we present some more examples of experimentally compressed and reconstructed images from the test dataset. The first row in Fig. S1 corresponds to the original images. The second and third row show the experimentally compressed and reconstructed images. The sizes of the images are shown in brackets. The PSNR/SSIM of the experimentally compressed images (from left to right) are 26.97 dB/0.94, 22.7 dB/0.85, 22.6 dB/0.81, 26.83 dB/0.88, 25.51 dB/0.84. The corresponding PSNR/SSIM of the JPEG compressed images are 28.86 dB/0.72, 27.34 dB/0.79, 26.93 dB/0.75 dB, 30.65 dB/0.87, 29.41 dB/0.87. For the compressed images, only one of the four compressed images are shown here. In Fig. S2 we show the histogram of PSNR and SSIM of all the reconstructed images from the test dataset that we compressed using our experimentally measured encoding matrix.

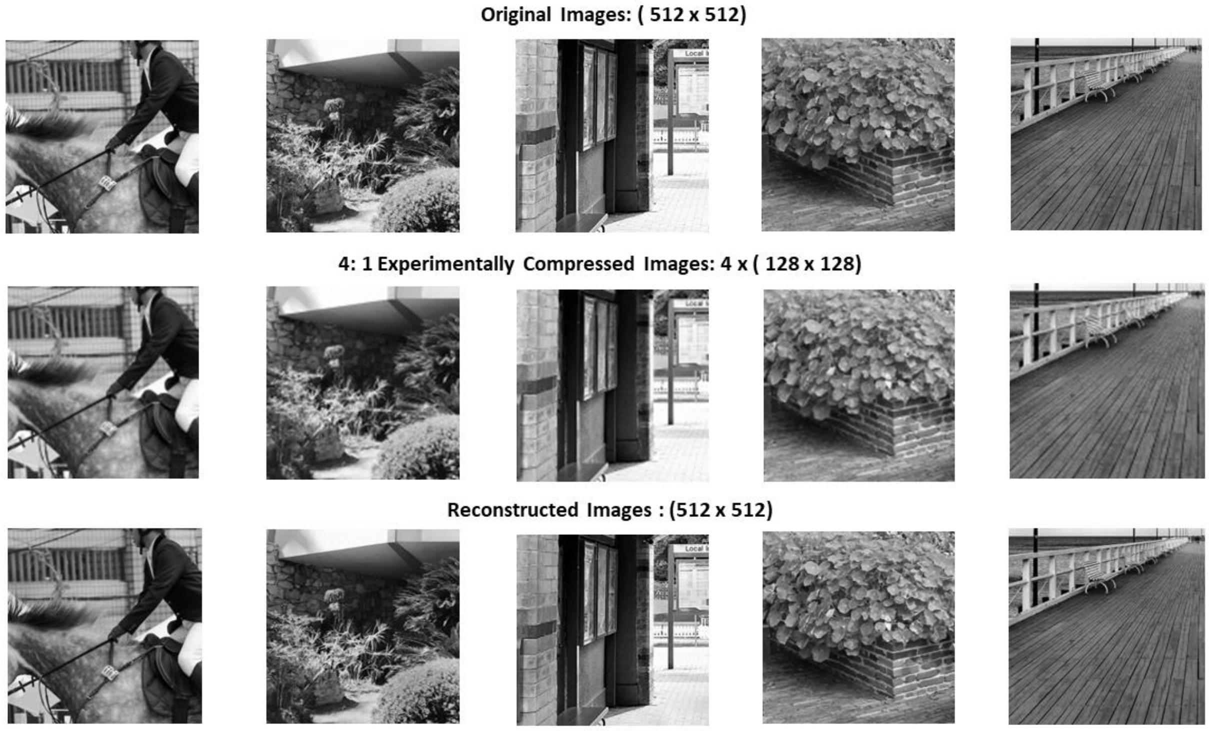

Figure S1: Examples of experimentally compressed images. The first row corresponds to original images taken from the test dataset. The second row are corresponding compressed images that are compressed using the experimentally measured encoding matrix. Only one of the 4 compressed images are shown. The third row corresponds to the reconstructed images. The sizes of the images are shown in brackets.

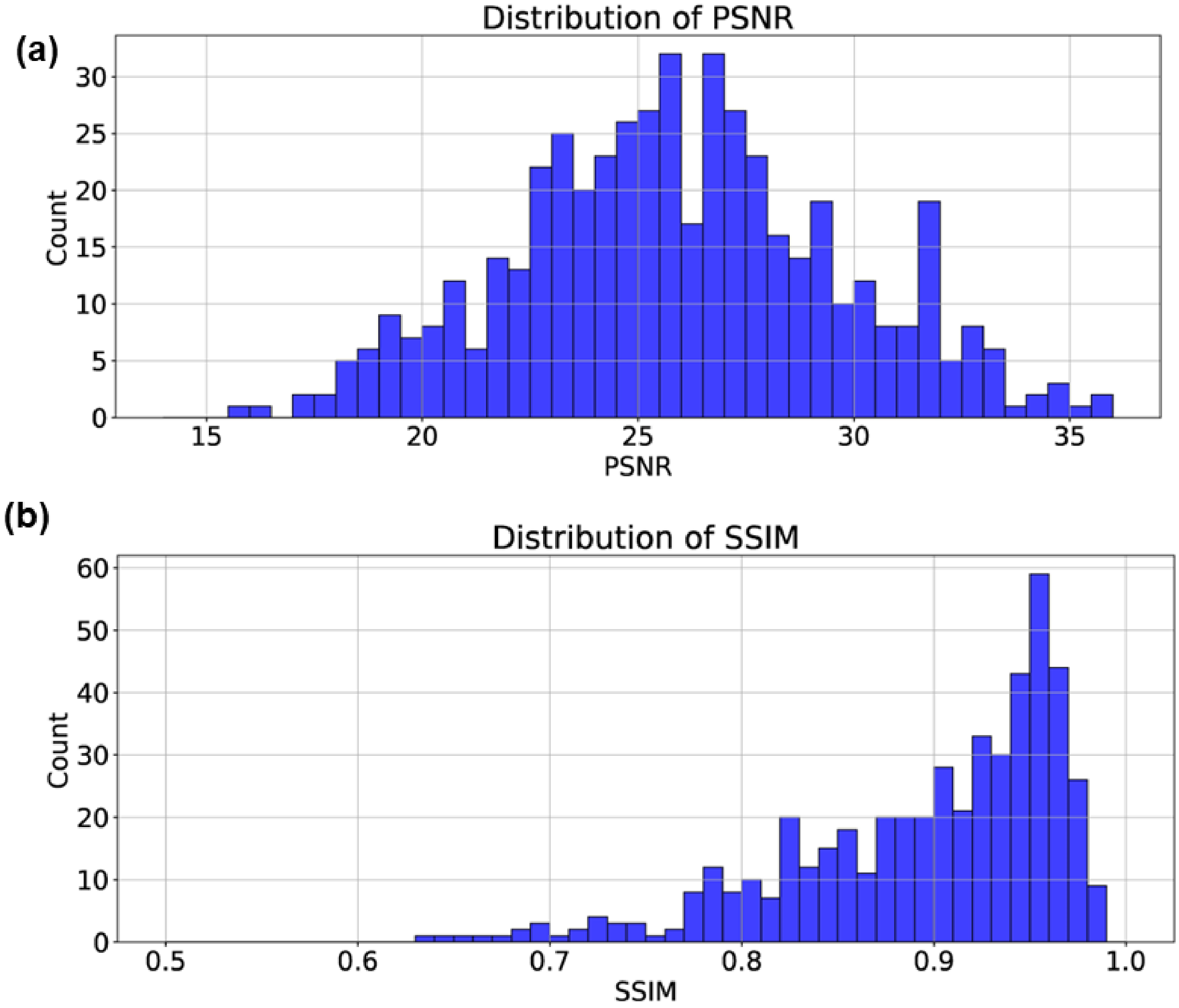

Figure S2: Statistics of reconstructed images that were compressed using experimentally measured encoding matrix. (a) Histogram of PSNR of all the reconstructed images from the test dataset. **(b)** Histogram of SSIM of all the reconstructed images from the test dataset.

S2. Energy consumption typical of mainstream electronic architectures

Today's digital accelerator landscape includes various parallel chip architectures with a range of core counts and performance metrics. Table 1 summarizes mainstream architectures and the energy consumption for these major categories.

Type	Name	Energy Efficiency
SoC	Apple A16 Bionic	0.4pJ/MAC [1]
	JPEG hardware codec	0.5~20pJ/MAC [2]
GPU	NVIDIA V100	4.6 pJ/MAC
	NVIDIA H100 SXM	0.7 pJ/MAC
ASIC	Google TPU v1	0.8 pJ/MAC
	Google TPU v4	1.2 pJ/MAC
FPGA	Xilinx Alveo U250	13.5 pJ/MAC
	Xilinx Versal VC2802	0.7 pJ/MAC
Desktop CPU	Intel i9-13900K	2.2nJ/MAC [3]
	AMD Ryzen 9 7950X	2.3nJ/MAC [4]

Table 1: Energy efficiency of prominent categories of digital accelerators. Typical energy efficiency of these digital accelerators is on the order of ~ 1 pJ/MAC.

S3. Integration of photonic encoder with silicon photonics and CMOS components

In the main text, we presented a passive, proof-of-concept device and showed experimental compression using a real-valued transmission matrix. While the passive photonic encoder forms the most critical component of the image processing engine, the complete optoelectronic image processing engine will require integration of active CMOS components such as modulators and detectors which will also enable operation using a complex-valued transmission matrix. This is because integration of modulators and detectors will allow us to extract both amplitude and phase of the encoded electric fields for different inputs using phase retrieval compressive measurement techniques. As shown in the main text, compression of images using complex transforms is more robust to experimental noise.

A high-level view of the photonic accelerator and electronic interface of our envisioned image processing engine is shown in Fig. S3. The optical components are inside the dotted box, and the CMOS electronic components are external to it. The optoelectronic accelerator is designed to convert N inputs into M outputs, where N and M can be different depending on the computing task. To convert data to the optical domain, a set of N modulators will be driven by analog signals that are transduced onto an optical carrier provided by a laser. Light then propagates through the accelerator device and is received by M photodetectors. The optical operation will be supported by multiple CMOS components: the driver sets the output voltage levels for the modulator, the transimpedance amplifier (TIA) converts the small current signal into a voltage and a secondary

amplifier sets the voltage level for the CMOS family. At either end of the link are the digital/analog (D/A) and analog/digital (A/D) converters.

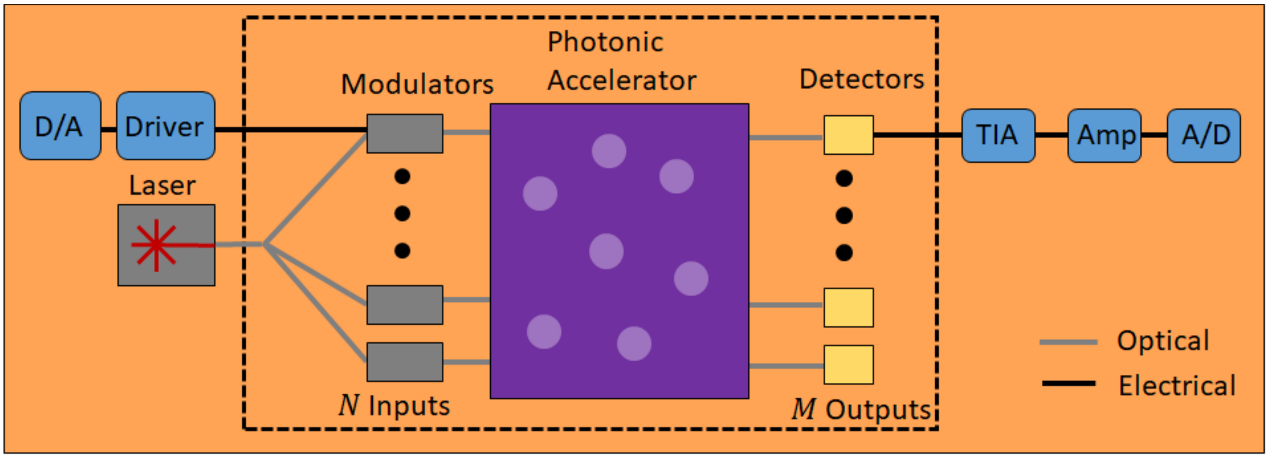

Figure S3. High-level view of the optoelectronic image processing engine. The complete image processing engine will include the photonic accelerator, modulators, detectors, and CMOS ICs. The silicon photonic components and CMOS components are placed inside and outside the dotted box, respectively. In general, a single laser (assumed to be off-chip here) could be used to drive multiple photonic accelerators.

For the active devices, which will have integrated detectors, we intend to use the Germanium (Ge) photodiodes currently being developed by the Sandia National Laboratories CMOS compatible silicon photonics process. As shown in Ref. 5, these photodiodes can be multimode with width ranging from 1.3 to 5.3 microns (i.e., can support from ~ 4 to ~20 modes at 1.55 microns). These photodiodes have a best-in-class 3 dB cutoff frequency of 45 GHz, responsivity of 0.8 A/W and dark current of 3 nA. In general, coupling efficiency in these waveguide integrated Ge detectors can be very high (> 90 %) without being limited by the intrinsic layer thickness of the device (see for example Ref. 6). In the next generation of data compression devices, we intend to further optimize coupling to the detectors by adiabatically tapering down the output silicon waveguides after the scattering region to match the number of modes supported by the Ge photodetector region.

Finally, we want to point out that for the energy calculations shown in the main text, we did account for coupling efficiency to the integrated photodetectors in our calculations. Our scattering medium provides a transmission of 30 % as shown in Section S5. However, we assumed 20 % overall transmission, which included ~67% transmission to the photodetectors.

S4. Denoising Images

As shown in the main text, our approach besides being robust to noise introduced in the analog photonic image compression step, also illustrates how this technique could be used for image denoising. From the perspective of the back-end image reconstruction neural network, noise added during the original image acquisition process (e.g., due to pixel noise, non-uniform responsivity, or simply low light levels) is equivalent to noise added during the image compression step. This case was explicitly tested and demonstrated in the main text using experimental data for noise. However, another potential complication can be that noise will be added during the data encoding step (i.e., on the high-speed integrated modulators). To confirm that our approach can also work well for such situations, i.e., can allow us to compress and denoise simultaneously, we performed simulations where we calculated the compression quality as a function of noise added to the input image (simulating the effect of noise introduced by the modulators while encoding the inputs).

We performed simulations for the case when different levels of noise (quantified in terms of SNR) are added to the original image data. Our denoising auto-encoder structure performs quite well even when noise is present in the original image data. Figures S4, S5, and S6, show examples of four images being compressed with different degrees of noise (SNR 10, 20, and 50 respectively) and the corresponding reconstructed images. As expected, because of the noise being added to the original image (top row of Figs. S4, S5, and S6), the compressed images (middle rows of the Figs.

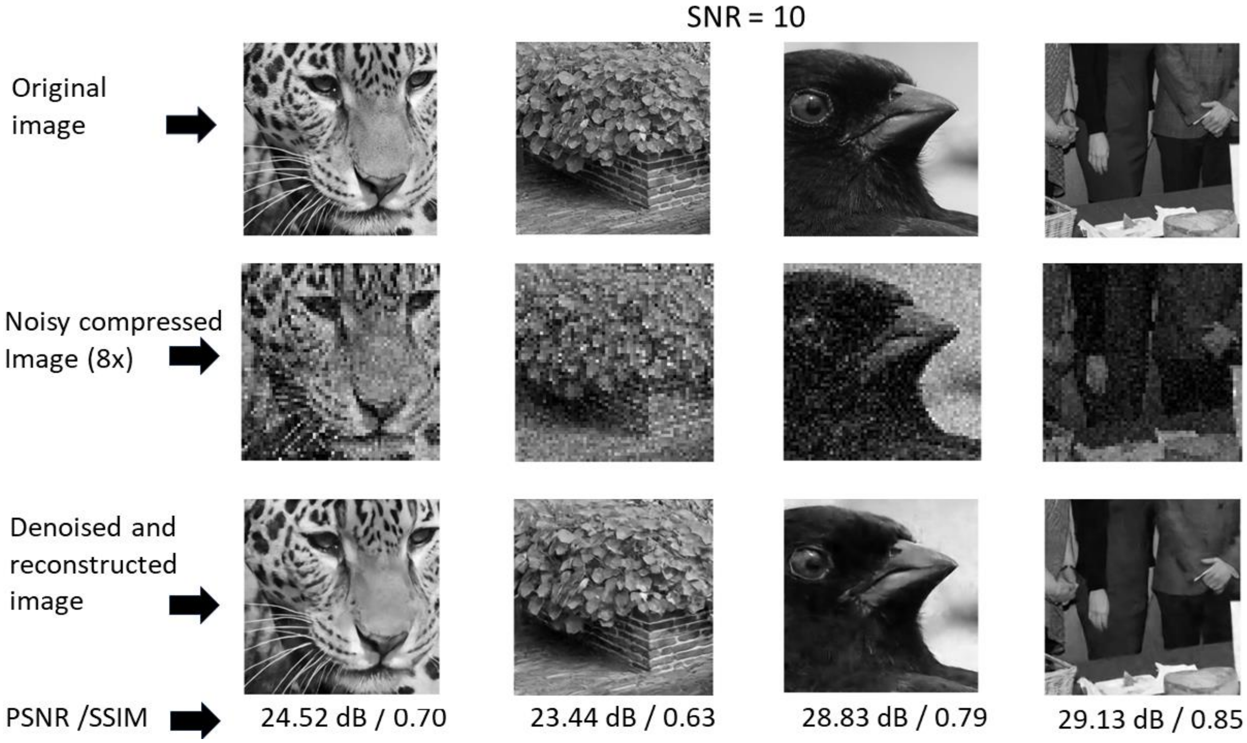

Figure S4. Examples of denoising and reconstruction of compressed images with added noise equivalent to SNR = 10. The first row corresponds to original images taken from the test dataset. The second row are corresponding compressed images that are compressed 8x using an 8x8 kernel. Only one of the 8 compressed images are shown. The images are compressed after adding gaussian noise to the original images shown in the first row. The third row corresponds to the denoised and reconstructed images with their corresponding PSNR and SSIM.

S4, S5, and S6), are noisy compared to the compressed images shown in Fig. 2 (b). Similar to the case shown in the main text, even for this case, the autoencoder network performs well in reconstructing and denoising the images. The denoised and reconstructed images along with their PSNR and SSIM are shown in the bottom row of the figures. Even with SNR as low as 10, we achieve PSNR > 20 dB and SSIM > 0.6 for most test images, comparable to state-of-the art image denoising techniques such as Wiener filtering, transform domain filtering methods, or convolutional neural network (CNN) based-methods [7].This confirms that our compression and denoising scheme works well regardless of whether noise is introduced during the original image acquisition stage or during the compression process.

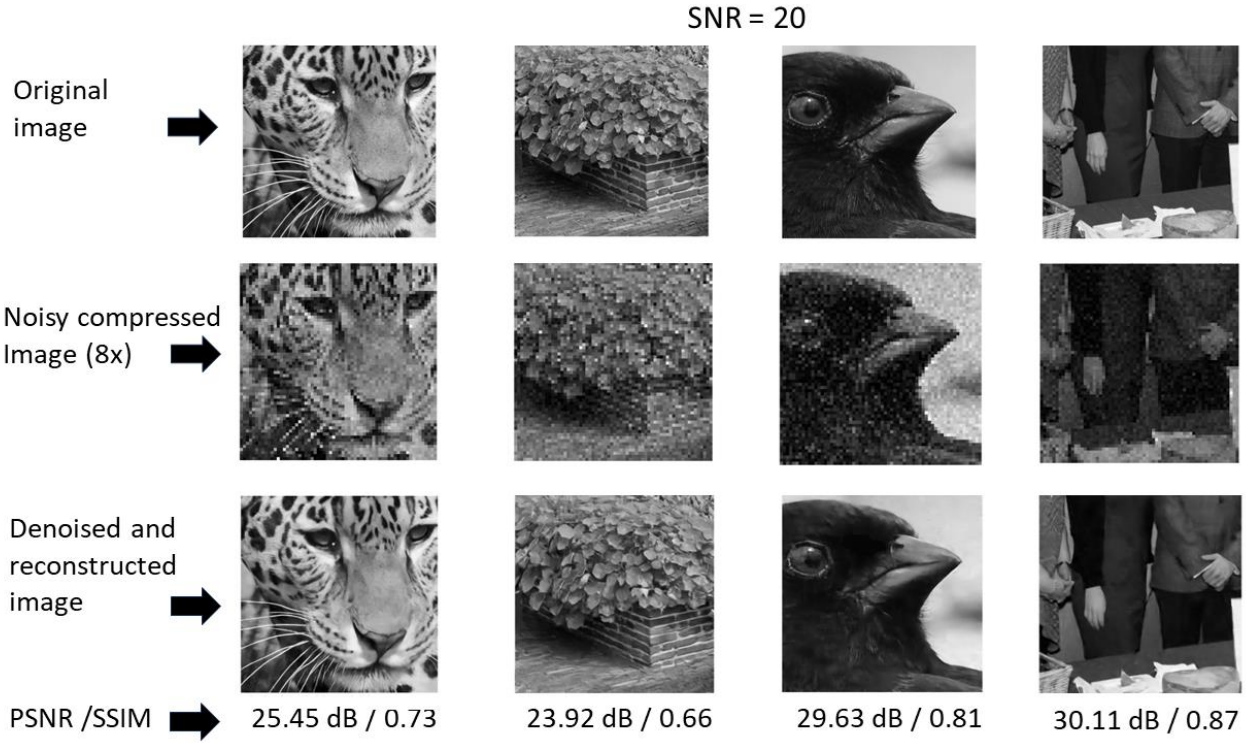

Figure S5. Examples of denoising and reconstruction of compressed images with added noise equivalent to SNR = 20. The first row corresponds to original images taken from the test dataset. The second row are corresponding compressed images that are compressed 8x using an 8x8 kernel. Only one of the 8 compressed images are shown. The images are compressed after adding gaussian noise to the original images shown in the first row. The third row corresponds to the denoised and reconstructed images with their corresponding PSNR and SSIM.

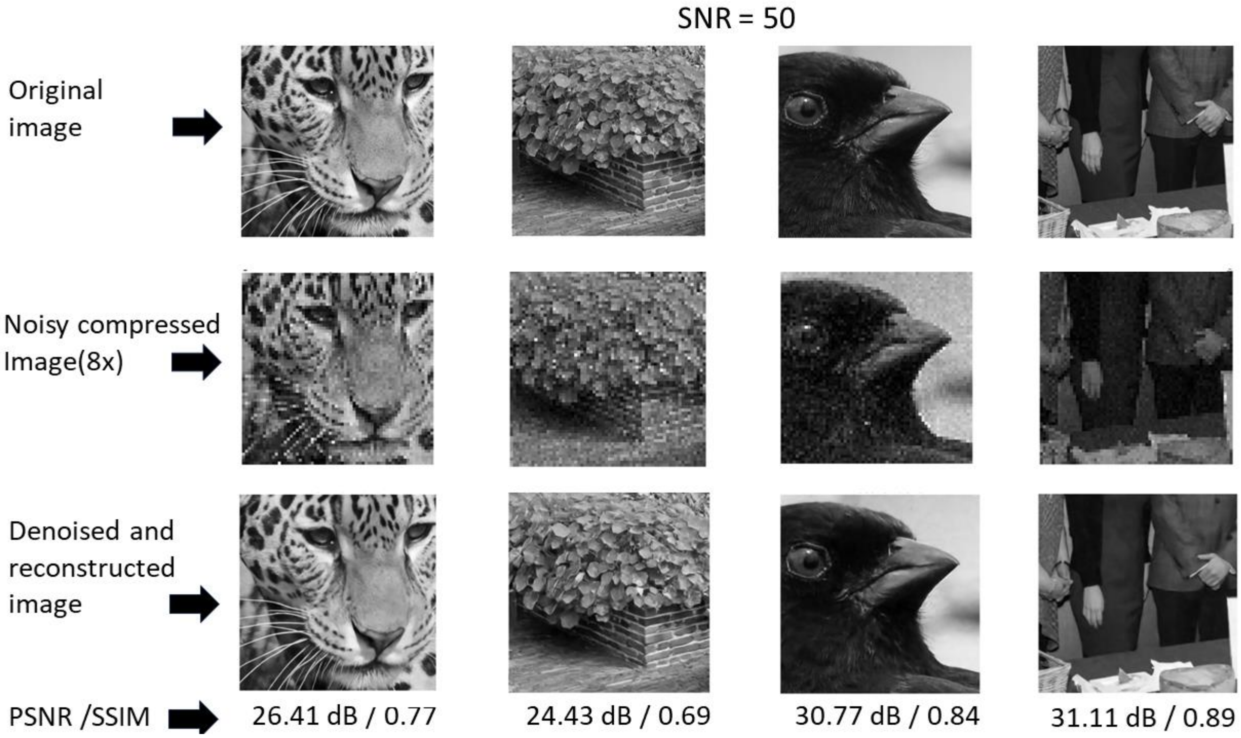

Figure S6. Examples of denoising and reconstruction of compressed images with added noise equivalent to SNR = 50. The first row corresponds to original images taken from the test dataset. The second row are corresponding compressed images that are compressed 8x using an 8x8 kernel. Only one of the 8 compressed images are shown. The images are compressed after adding gaussian noise to the original images shown in the first row. The third row corresponds to the denoised and reconstructed images with their corresponding PSNR and SSIM.

S5. Additional Experimental Characterization

Long-term stability measurements of the photonic encoder.

In the main text, we performed two sequential measurements of the transmission matrix of the photonic encoder to determine the experimental noise and demonstrate both compression and denoising. The noise determined by these experiments was related to the noise introduced by the laser and electronics and is not related to fluctuations or noise introduced by the photonic encoder. In fact, our photonic encoder, being on a silicon chip, is extremely stable and does not require thermal stabilization to operate at room temperature. To confirm this, we performed long-term stability measurements of the photonic encoder where we coupled light from a narrowband coherent laser into one of the input waveguides and recorded the transmitted speckle pattern every 30 seconds for a total of 60 hours. Figure S7 (a) shows the experimental setup used for this measurement and Fig S7 (b) shows the raw data. As shown in Fig. S7 (b), we noticed that the output speckle pattern remains unchanged except for some jitter that is observed due to microscope alignment and coupling and/or drift of the coupling optics. After performing image registration (i.e. performing a cross-correlation of the speckle pattern to see how much this pattern shifted, and then shifting to compensate) to compensate for the drifts of the optical components, we see

extremely minimal changes in the output speckle pattern confirming that our photonic encoder is extremely stable and can be utilized for image compression at room temperatures under standard lab conditions without requiring additional thermal stabilization schemes. We stopped this measurement after 60 hours due to practical reasons.

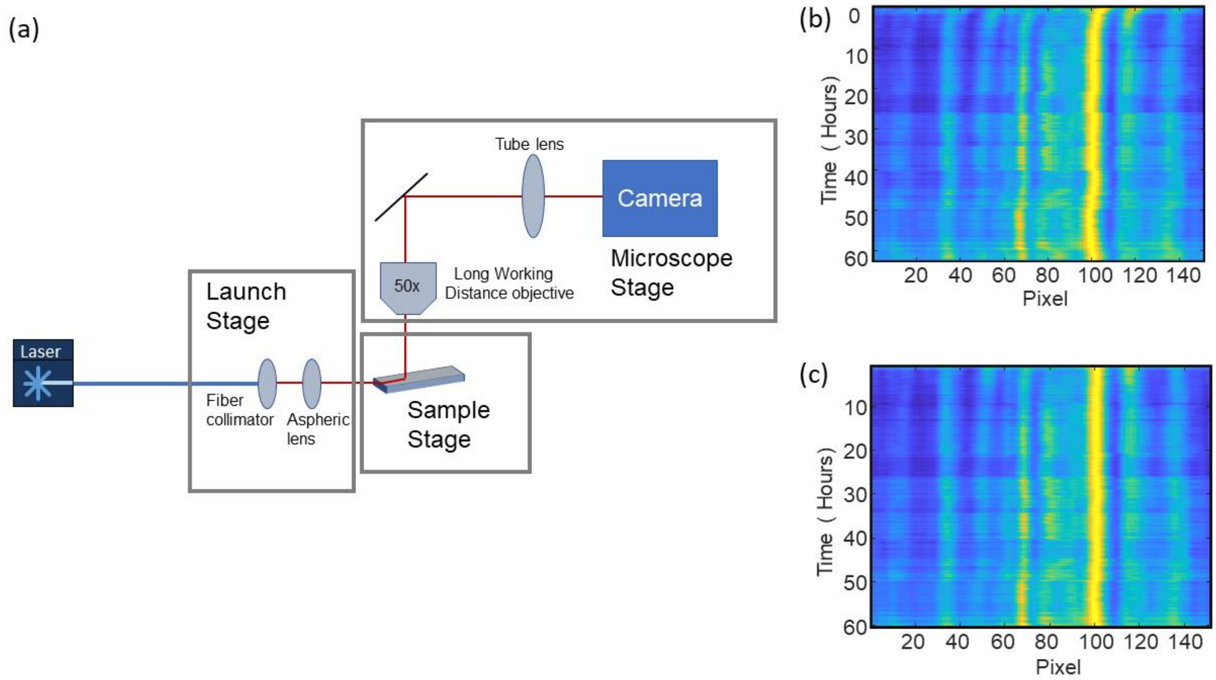

Figure S7. Experimental characterization of long-term stability of the photonic encoder. (a) Experimental setup used to measure and record the speckle pattern to confirm long-term stability of the photonic encoder. Light from a narrow band laser is coupled to one of the input waveguides and the transmitted speckle pattern is imaged and recorded (similar to results shown in Fig. 5 and 6 of the main text) every 30 seconds for a span of 60 hours using a camera. **(b)** The raw data corresponding to intensities of the output speckle pattern at different time steps is shown. The data is extracted from images similar to ones shown in Fig. 5(d) of the main text. The x-axis of the data corresponds to the pixel number at the output and y-axis corresponds to the time steps in hours. **(c)** The processed data corresponding to intensities of the output speckle pattern at different time steps is shown. Data processing involved performing image registration (i.e., performing a cross-correlation of the speckle pattern to see how much this pattern shifted, and then shifting to compensate) to compensate for the drifts of the optical components.

Determination of transmission through the photonic encoder.

In the main text, we performed calculations to compare the energy efficiency of our approach to digital electronic approaches for compression. One of the important parameters that determine the energy efficiency of our photonic approach for compression is the loss or transmission through the photonic encoder. To determine experimentally the transmission through the photonic encoder, we used a broadband amplified spontaneous emission (ASE) light source to couple light into the input waveguides of the photonic encoder and imaged the out-of-plane scattered light from the photonic

encoder to determine the spatial distribution of the intensity pattern within the photonic encoder. Fig. S8 (a) shows the experimental setup used for this measurement. Unlike a narrow band coherent laser source used for the other measurements, a broadband ASE source helps us to reduce speckle contrast of the intensity pattern within the photonic encoder which, as shown later, helps us in getting more accurate loss measurements. The light from the ASE source is coupled to each waveguide and the out-of-plane scattered light from the photonic encoder is imaged and recorded using a camera. The measurement is repeated for each of 16 input waveguides and each image was first normalized by average scattered power to compensate for waveguide coupling variation. The 16 images are then digitally added to get an average two-dimensional intensity pattern of the photonic encoder. The resulting image is shown in Figure S8 (b).

As shown in many of our previous works [8-10], for photonic structures with randomly positioned scattering centers, the cross-section (along *y*) integrated intensity within the photonic structures that is obtained by imaging the out-of-plane scattered light is proportional to the flux transmitted through the photonic structure. We therefore integrated the intensity of the image shown in Fig. S8 (b) along the cross-section (*y*) and in Fig. S8 (c) we plot the cross-section integrated intensity along the propagation direction (*x*). The cross-section integrated intensity within the photonic encoder, as expected by Fick's law of diffusive light transport [8-10], decays linearly along the propagation direction and from the linear decay (shown by the red solid line in Fig. S8 (c)), we can estimate the transmission through the photonic encoder. For the photonic encoder used for this work the measured transmission is ~ 34.7 %.

We want to point here that for this work, we fabricated photonic encoders with different scattering strengths and dimensions. We measured empirically the losses through the different photonic encoders and optimized the scattering strength and geometry such that we achieve cross-section integrated intensity within the photonic encoder that decays linearly as opposed to an exponential decay which indicates much more significant loss.

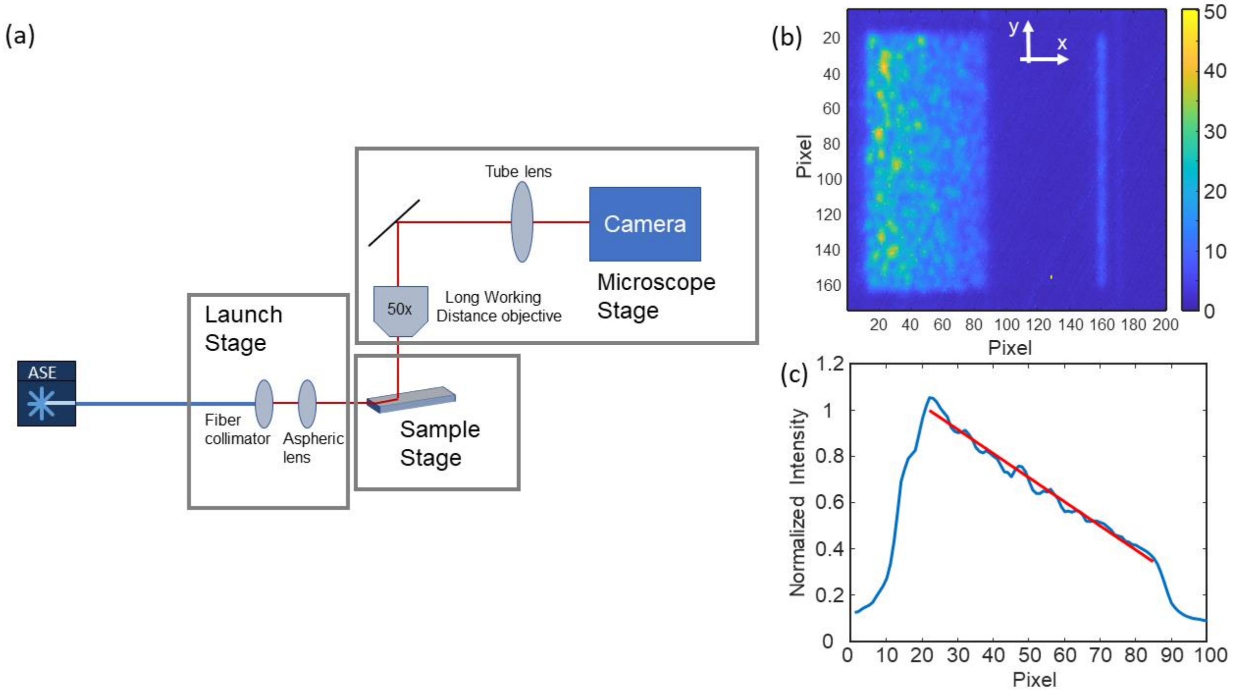

Figure S8. **Experimental characterization of transmission through the photonic encoder.** **(a)** Experimental setup used to measure and record the intensity pattern within the photonic encoder to determine the transmission through the photonic encoder. Light from a broadband ASE light source is coupled to one of the input waveguides and the intensity pattern within the photonic encoder is imaged and recorded. This measurement is repeated for all the 16 input waveguides. **(b)** The averaged intensity pattern within the photonic encoder that is obtained by averaging 16 images which are obtained by coupling light into the 16 input waveguides one at a time. The *x*-axis of the image corresponds to light propagation direction and *y*-axis corresponds to the cross-section of the photonic encoder. **(c)** The cross-section integrated intensity within the photonic encoder plotted along the propagation direction *x*. The red solid line shows the linear fit to the linear decay of the flux within the photonic encoder.

S6. Comparison of Digital JPEG and Photonic Compression

In Figure 3(c, d) of the main text, the image that we used as an example could only be compressed up to 1:45 using the JPEG algorithm. Below we compare compression ratio of 1: 64 for JPEG compression and our photonic approach for more test images. For a fair comparison of JPEG to the photonic approach (which allows fixed compression ratio of 1:64 for all images), below we only present images which can be compressed to 1:64 using the JPEG algorithm.

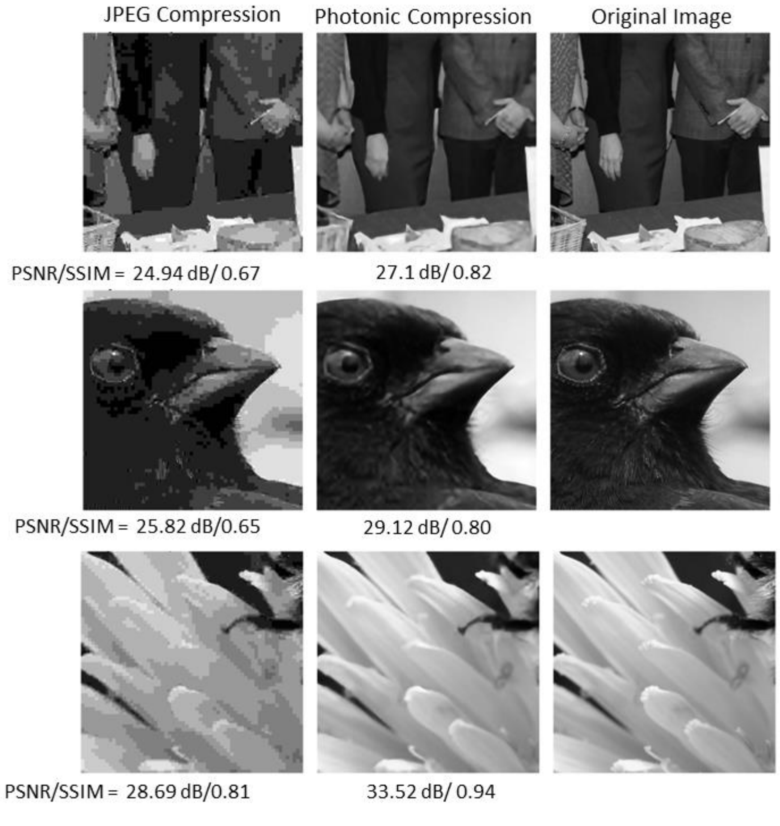

Figure S9. Comparison of photonic image compression to digital JPEG image compression. Comparison of reconstructed images that were compressed using the photonic compression approach using an 8x8 kernel with compression ratio of 1:64 to digital JPEG compression with compression ratio of 1:64. The original test images are also shown and the corresponding PSNR/SSIM of both the approaches are stated along with the images.

References

[1] Apple A16 Bionic Processor - Benchmarks and Specs - NotebookCheck.net Tech Typical performance: 3.6W (average), 5W (max). Architecture: 2 "Everest" 3.46 GHz ARM cores, 4 "Sawtooth" 2.02 GHz ARM cores, 16-core neural engine with a total of 17 TOPS throughput (16 16×16 systolic arrays @ 3.46GHz clock rate), unspecified throughput of photonic engine.

[2] Turcza, P., "Entropy encoder for low-power low-resources high-quality CFA image compression." *Signal Processing: Image Communication* **106**, 116716 (2022).

[3] Intel® Core™ i9-13900K Processor 253W, dual threads per core, 8 cores at 5.8GHz clock speed, 16 cores at 4.3GHz clock speed.

[4] AMD Ryzen™ 9 7950X Desktop Processors | AMD 170W, dual threads per core, 16 cores at 4.5GHz clock speed.

[5] DeRose, C. T. et. al., "Ultra compact 45 GHz CMOS compatible Germanium waveguide photodiode with low dark current", *Optics Express* **19 (25)**, 24897-24904 (2011).

[6] Ahn, D. et al, "High performance, waveguide integrated Ge photodetectors", *Optics Express* **15(7)**, 3916-3921 (2007).

[7] Fan, L., Zhang, F., Fan, H., Zhang, C., "Brief review of image denoising techniques", *Visual computing for industry, biomedicine, and art* **2**, 7 (2019).

[8] Yamilov, A., Sarma, R., Redding, B., Payne, B., Noh, H., Cao, H. "Position-dependent diffusion of light in disordered waveguides." *Physical Review Letters* **112**, 023904 (2014).

[9] Sarma, R., Golubev, T., Yamilov, A., Cao, H. "Control of light diffusion in a disordered photonic waveguide." *Applied Physics Letters* **105**, 041104 (2014).

[10] Sarma, R., Yamilov, A. G., Petrenko, S., Bromberg, Y., Cao, H. "Control of energy density inside a disordered medium by coupling to open or closed channels." *Physical Review Letters* **117**, 086803 (2016).
